# Supplementary material for: Experimental Data Driven AI Framework for Flexible Protein Conformational Reconstruction
Source: bioRxiv. 2026 Mar 14:2026.03.12.708611. Preprint. [Version 1] doi: 10.64898/2026.03.12.708611 (PMC13060840; doi:10.64898/2026.03.12.708611)
Supplement: Supplement 1 [file media-1.pdf]

Supplementary Figure - Page 1/20

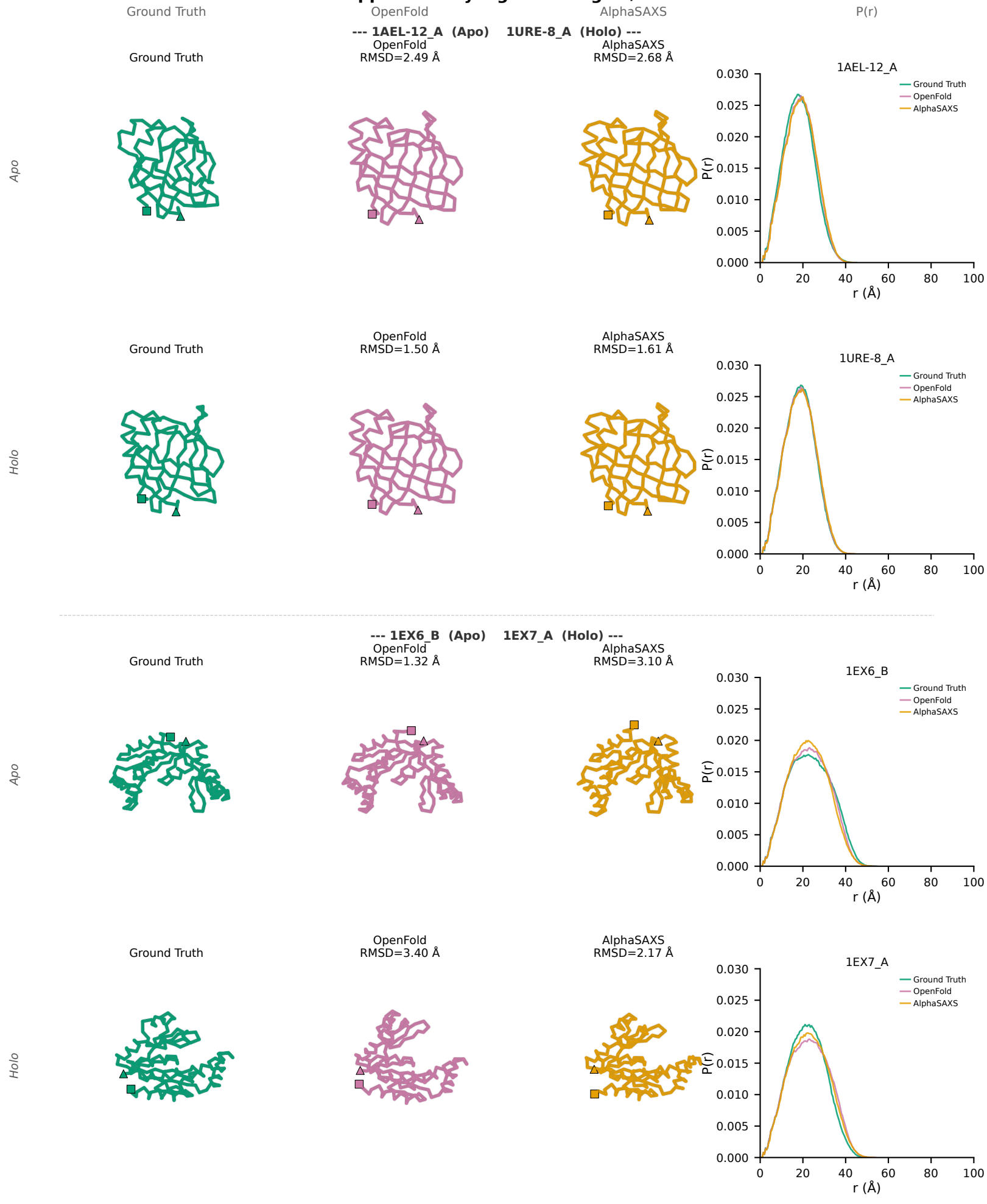

Ground Truth OpenFold AlphaSAXS P(r)

--- 1F3Y-17_A (Apo) 1JKN-15_A (Holo) ---

Apo

Ground Truth

OpenFold
RMSD=3.32 Å

AlphaSAXS
RMSD=5.41 Å

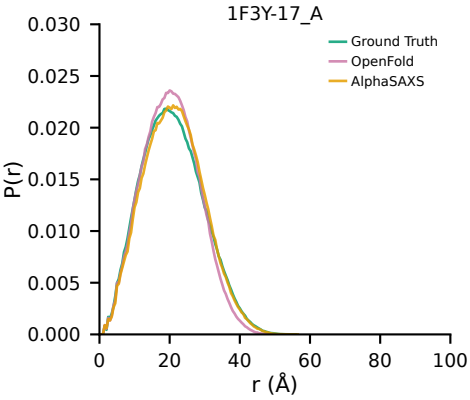

Holo

Ground Truth

OpenFold
RMSD=2.73 Å

AlphaSAXS
RMSD=4.15 Å

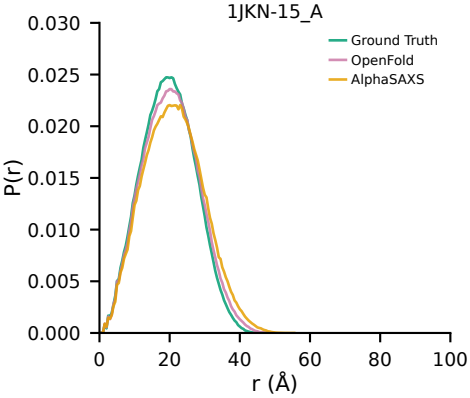

--- 1FMF-4_A (Apo) 1ID8-11_A (Holo) ---

Apo

Ground Truth

OpenFold
RMSD=17.03 Å

AlphaSAXS
RMSD=15.70 Å

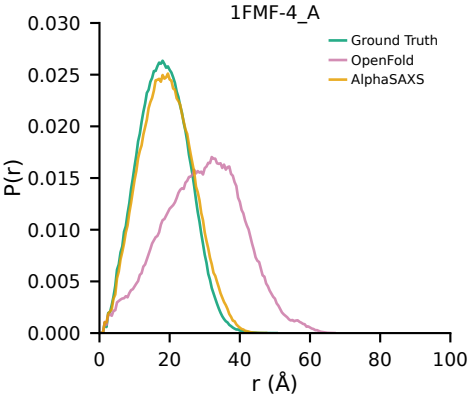

Holo

Ground Truth

OpenFold
RMSD=17.52 Å

AlphaSAXS
RMSD=16.62 Å

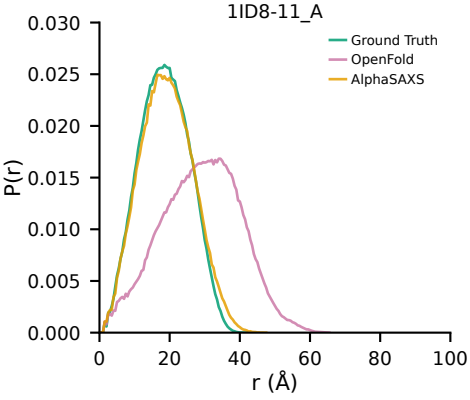

Ground Truth | OpenFold | AlphaSAXS | P(r)

--- 1GH1-7_A (Apo) 1CZ2-8_A (Holo) ---

Apo

Ground Truth

OpenFold
RMSD=2.54 Å

AlphaSAXS
RMSD=2.32 Å

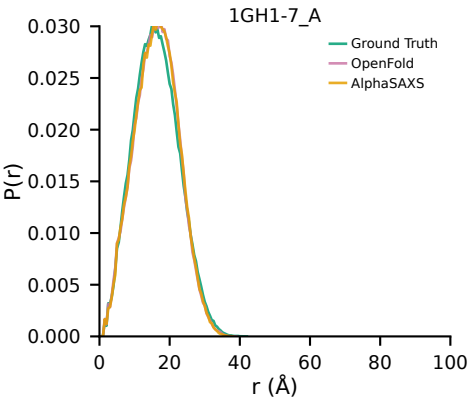

Holo

Ground Truth

OpenFold
RMSD=2.69 Å

AlphaSAXS
RMSD=2.43 Å

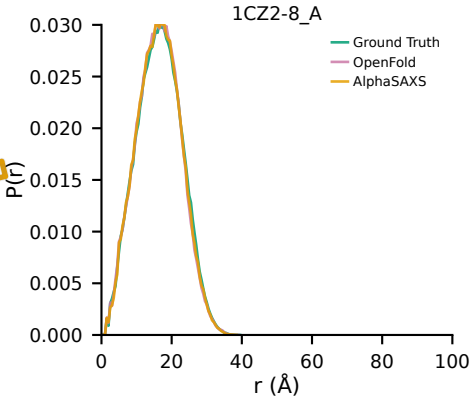

--- 1IJA-15_A (Apo) 2KID-4_A (Holo) ---

Apo

Ground Truth

OpenFold
RMSD=3.42 Å

AlphaSAXS
RMSD=4.38 Å

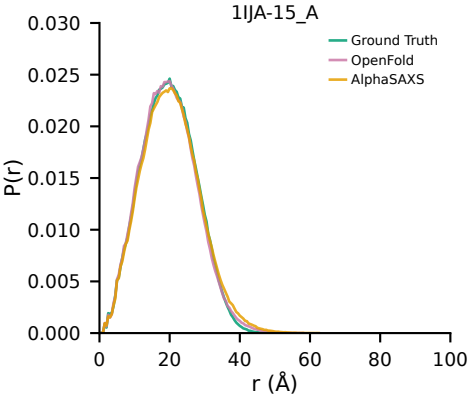

Holo

Ground Truth

OpenFold
RMSD=2.34 Å

AlphaSAXS
RMSD=1.89 Å

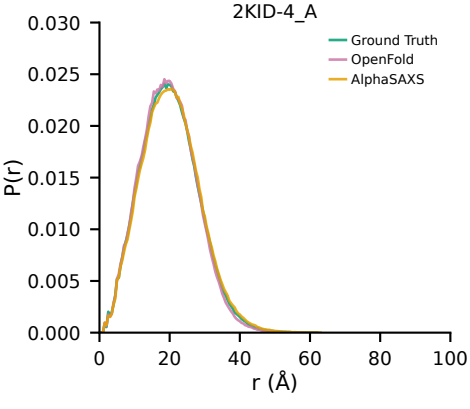

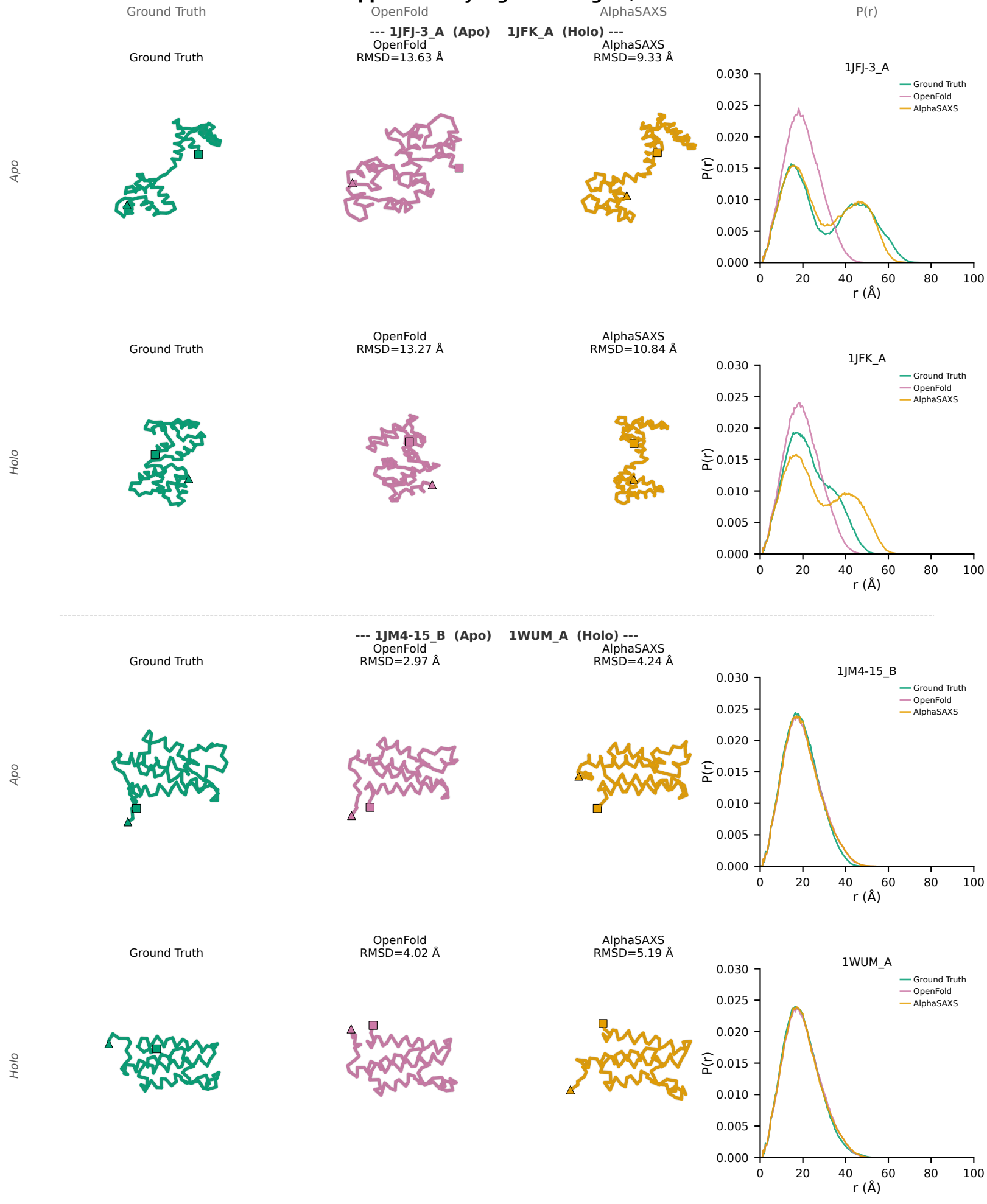
Ground Truth
OpenFold
AlphaSAXS
P(r)
--- 1JFJ-3_A (Apo) 1JFK_A (Holo) ---
Apo
Holo
Ground Truth
OpenFold
RMSD=13.63 Å
AlphaSAXS
RMSD=9.33 Å
1JFJ-3_A
Ground Truth
OpenFold
AlphaSAXS
P(r)
r (Å)
Ground Truth
OpenFold
RMSD=13.27 Å
AlphaSAXS
RMSD=10.84 Å
1JFK_A
--- 1JM4-15_B (Apo) 1WUM_A (Holo) ---
Ground Truth
OpenFold
RMSD=2.97 Å
AlphaSAXS
RMSD=4.24 Å
1JM4-15_B
Ground Truth
OpenFold
RMSD=4.02 Å
AlphaSAXS
RMSD=5.19 Å
1WUM_A

Ground Truth OpenFold AlphaSAXS P(r)

--- 1K2H-4_A (Apo) 1ZFS-13_B (Holo) ---

Apo

Ground Truth

OpenFold
RMSD=6.55 Å

AlphaSAXS
RMSD=6.28 Å

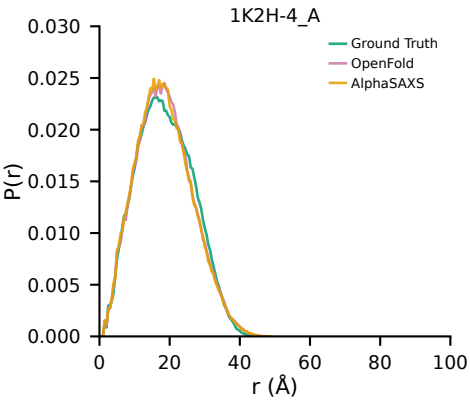

Holo

Ground Truth

OpenFold
RMSD=2.11 Å

AlphaSAXS
RMSD=2.56 Å

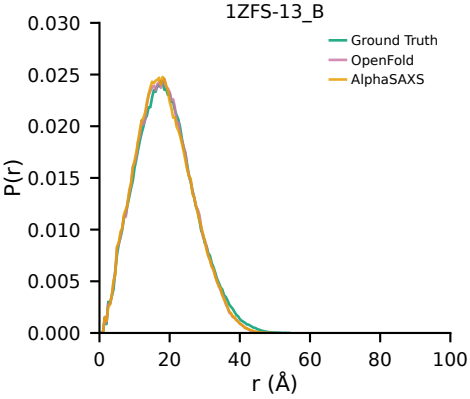

--- 1LIP-2_A (Apo) 1JTB-6_A (Holo) ---

Apo

Ground Truth

OpenFold
RMSD=2.29 Å

AlphaSAXS
RMSD=1.87 Å

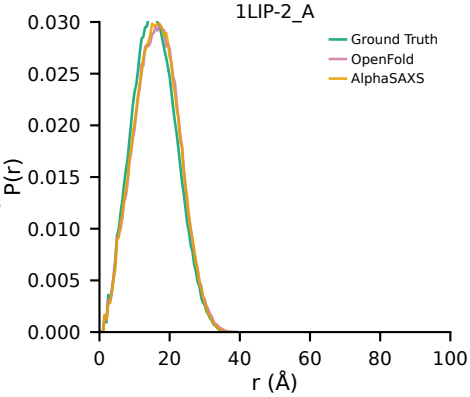

Holo

Ground Truth

OpenFold
RMSD=2.94 Å

AlphaSAXS
RMSD=2.68 Å

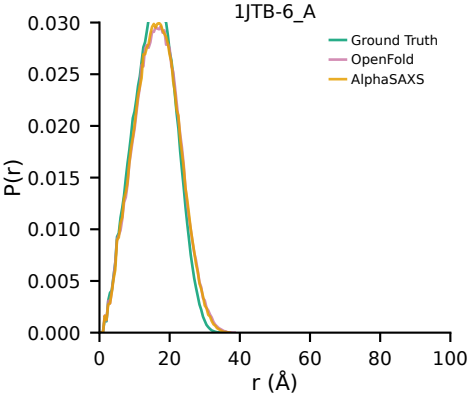

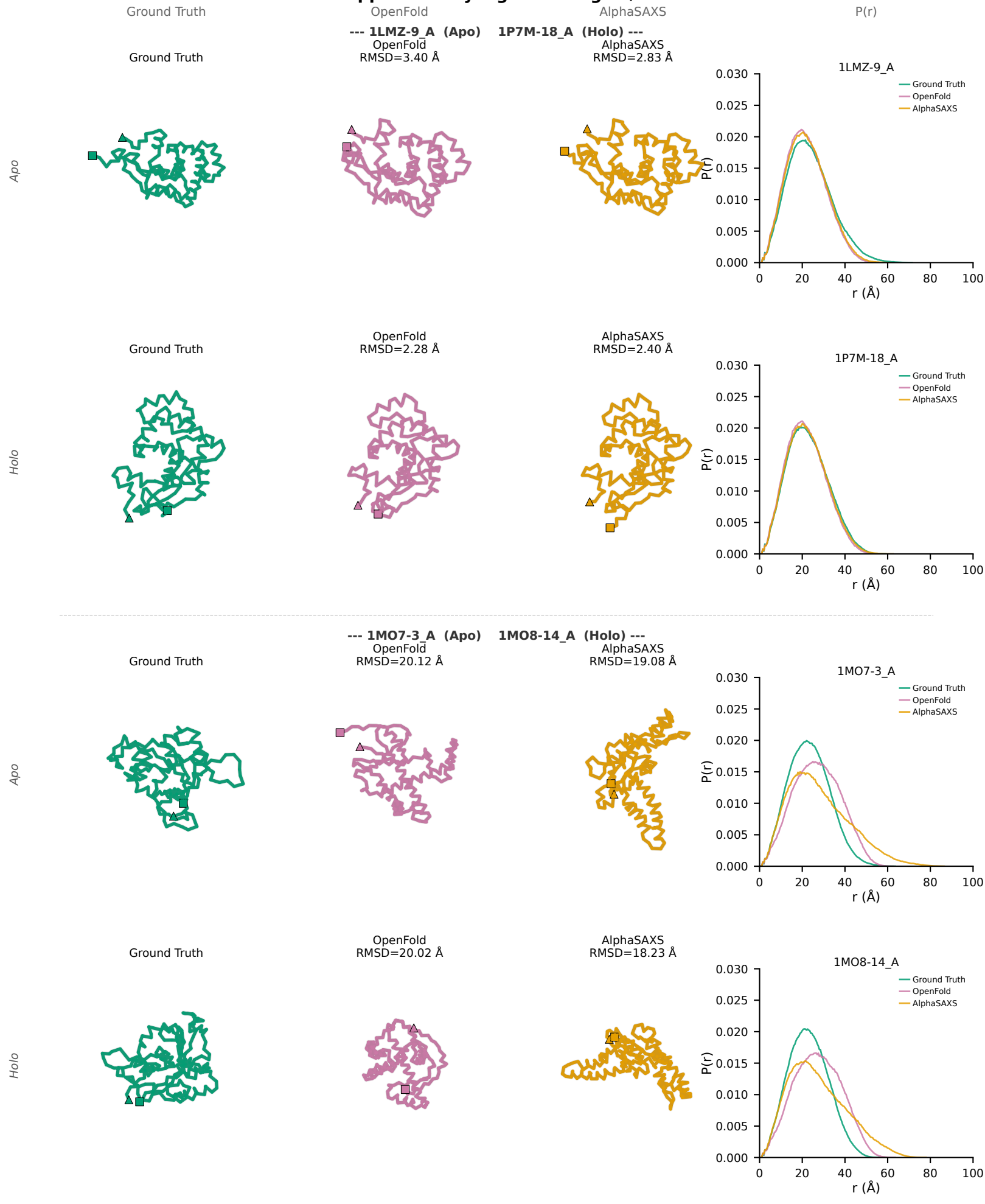
Ground Truth
OpenFold
AlphaSAXS
P(r)
--- 1LMZ-9_A (Apo) 1P7M-18_A (Holo) ---
Apo
Holo
Ground Truth
OpenFold
RMSD=3.40 Å
AlphaSAXS
RMSD=2.83 Å
1LMZ-9_A
Ground Truth
OpenFold
AlphaSAXS
P(r)
r (Å)
OpenFold
RMSD=2.28 Å
AlphaSAXS
RMSD=2.40 Å
1P7M-18_A
--- 1MO7-3_A (Apo) 1MO8-14_A (Holo) ---
OpenFold
RMSD=20.12 Å
AlphaSAXS
RMSD=19.08 Å
1MO7-3_A
OpenFold
RMSD=20.02 Å
AlphaSAXS
RMSD=18.23 Å
1MO8-14_A

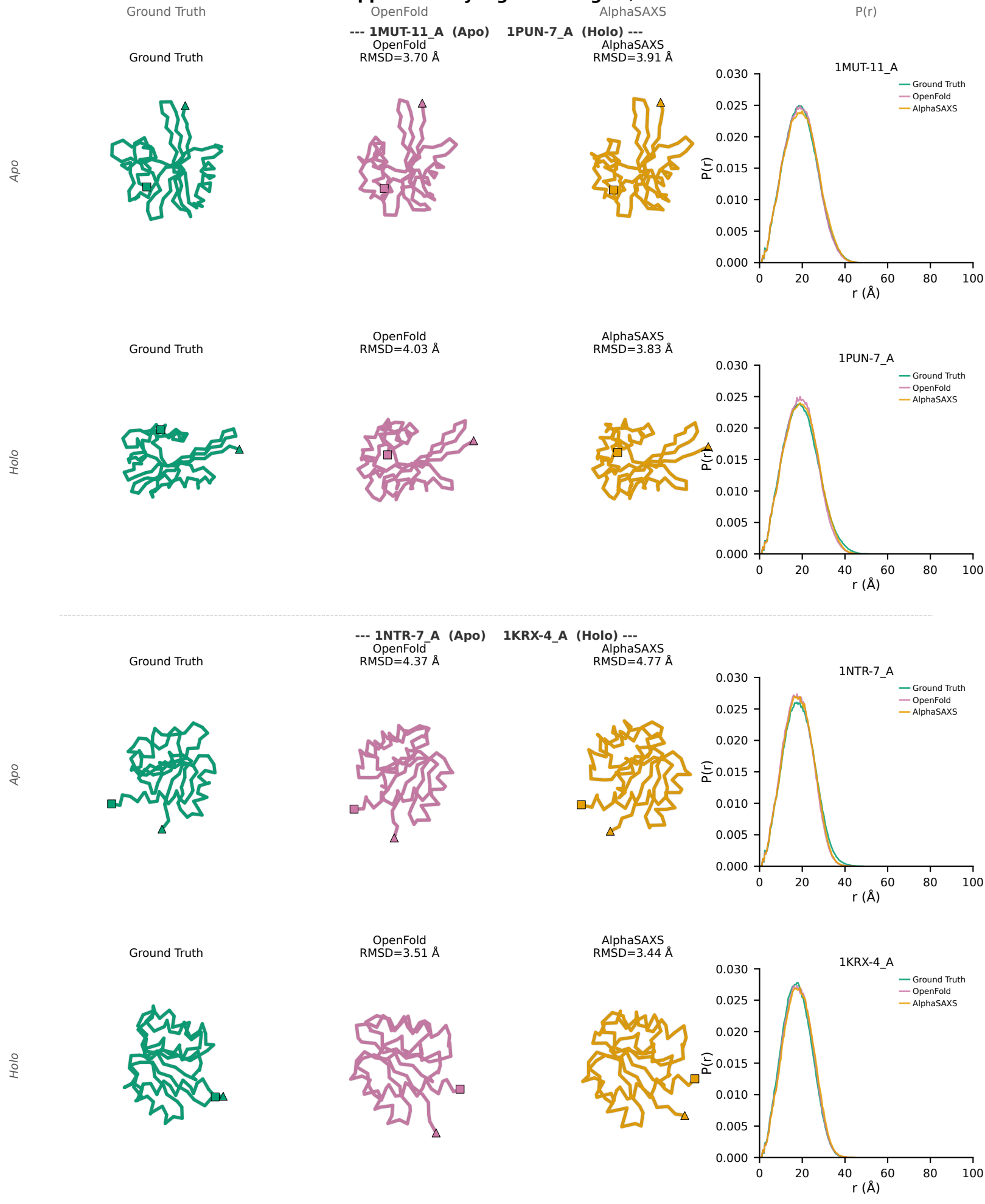
Ground Truth
OpenFold
AlphaSAXS
P(r)
--- 1MUT-11_A (Apo) 1PUN-7_A (Holo) ---
Apo
Holo
OpenFold
RMSD=3.70 Å
AlphaSAXS
RMSD=3.91 Å
1MUT-11_A
OpenFold
RMSD=4.03 Å
AlphaSAXS
RMSD=3.83 Å
1PUN-7_A
--- 1NTR-7_A (Apo) 1KRX-4_A (Holo) ---
OpenFold
RMSD=4.37 Å
AlphaSAXS
RMSD=4.77 Å
1NTR-7_A
OpenFold
RMSD=3.51 Å
AlphaSAXS
RMSD=3.44 Å
1KRX-4_A
Ground Truth
OpenFold
AlphaSAXS
P(r)
r (Å)

Ground Truth | OpenFold | AlphaSAXS | P(r)

--- 1ORM-9_A (Apo) 1QJ8_A (Holo) ---

Apo

Ground Truth

OpenFold
RMSD=5.65 Å

AlphaSAXS
RMSD=5.59 Å

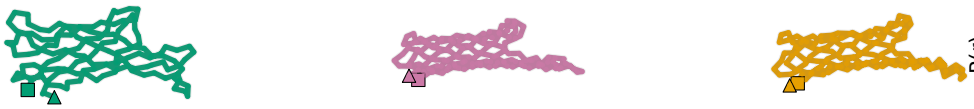

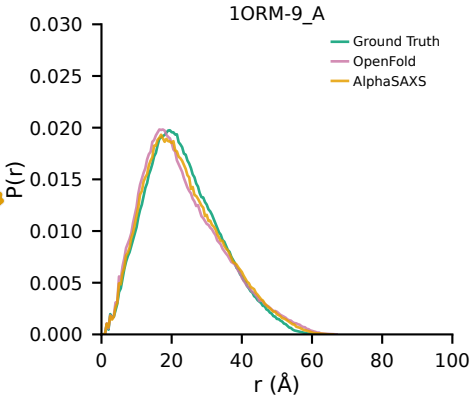

Holo

Ground Truth

OpenFold
RMSD=1.28 Å

AlphaSAXS
RMSD=2.86 Å

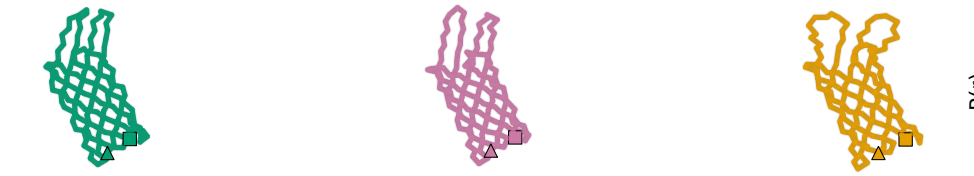

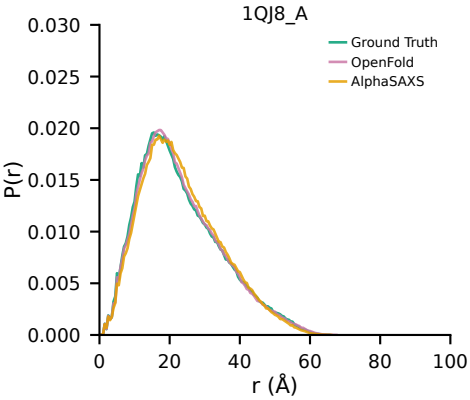

--- 1S2O_A (Apo) 1TJ5_A (Holo) ---

Apo

Ground Truth

OpenFold
RMSD=3.11 Å

AlphaSAXS
RMSD=2.19 Å

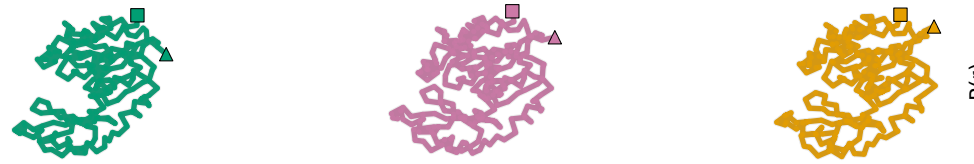

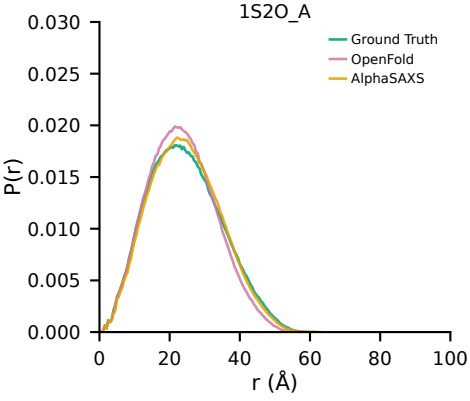

Holo

Ground Truth

OpenFold
RMSD=0.59 Å

AlphaSAXS
RMSD=1.85 Å

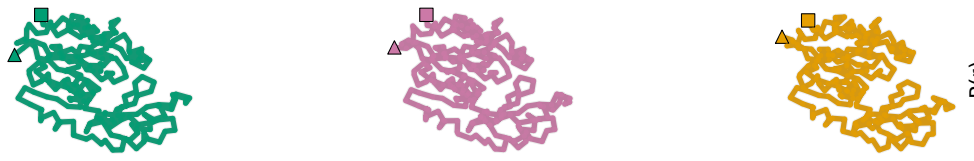

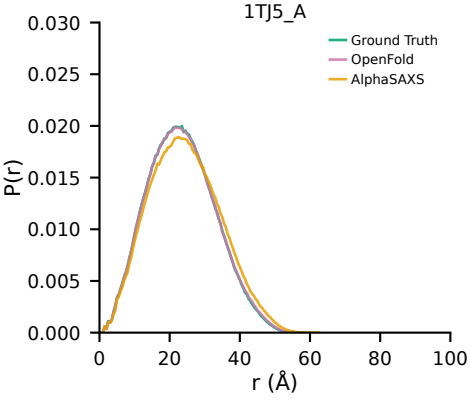

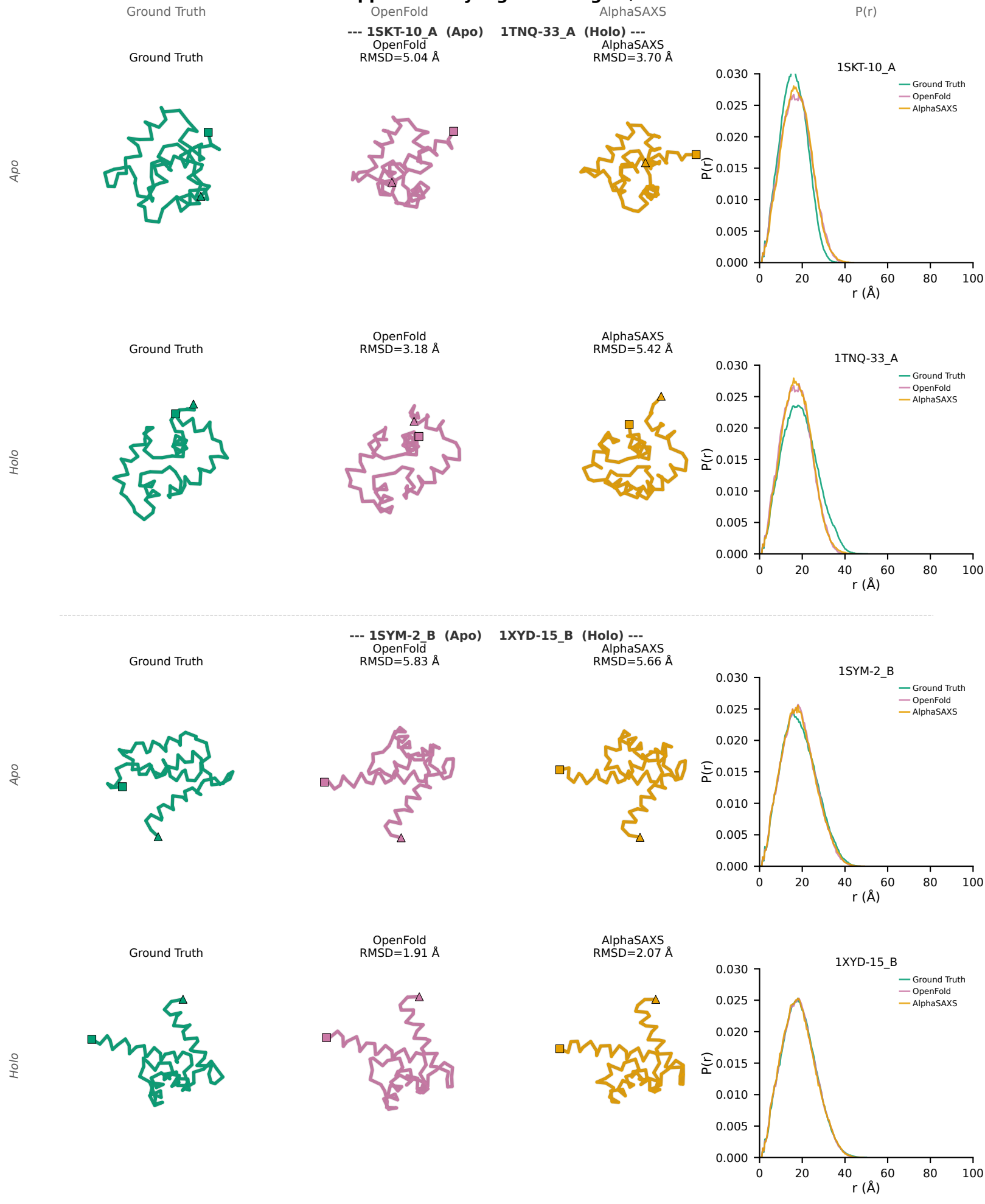
Ground Truth
OpenFold
AlphaSAXS
P(r)
--- 1SKT-10_A (Apo) 1TNQ-33_A (Holo) ---
Apo
Holo
Ground Truth
OpenFold
RMSD=5.04 Å
AlphaSAXS
RMSD=3.70 Å
1SKT-10_A
Ground Truth
OpenFold
AlphaSAXS
P(r)
r (Å)
Ground Truth
OpenFold
RMSD=3.18 Å
AlphaSAXS
RMSD=5.42 Å
1TNQ-33_A
--- 1SYM-2_B (Apo) 1XYD-15_B (Holo) ---
OpenFold
RMSD=5.83 Å
AlphaSAXS
RMSD=5.66 Å
1SYM-2_B
OpenFold
RMSD=1.91 Å
AlphaSAXS
RMSD=2.07 Å
1XYD-15_B

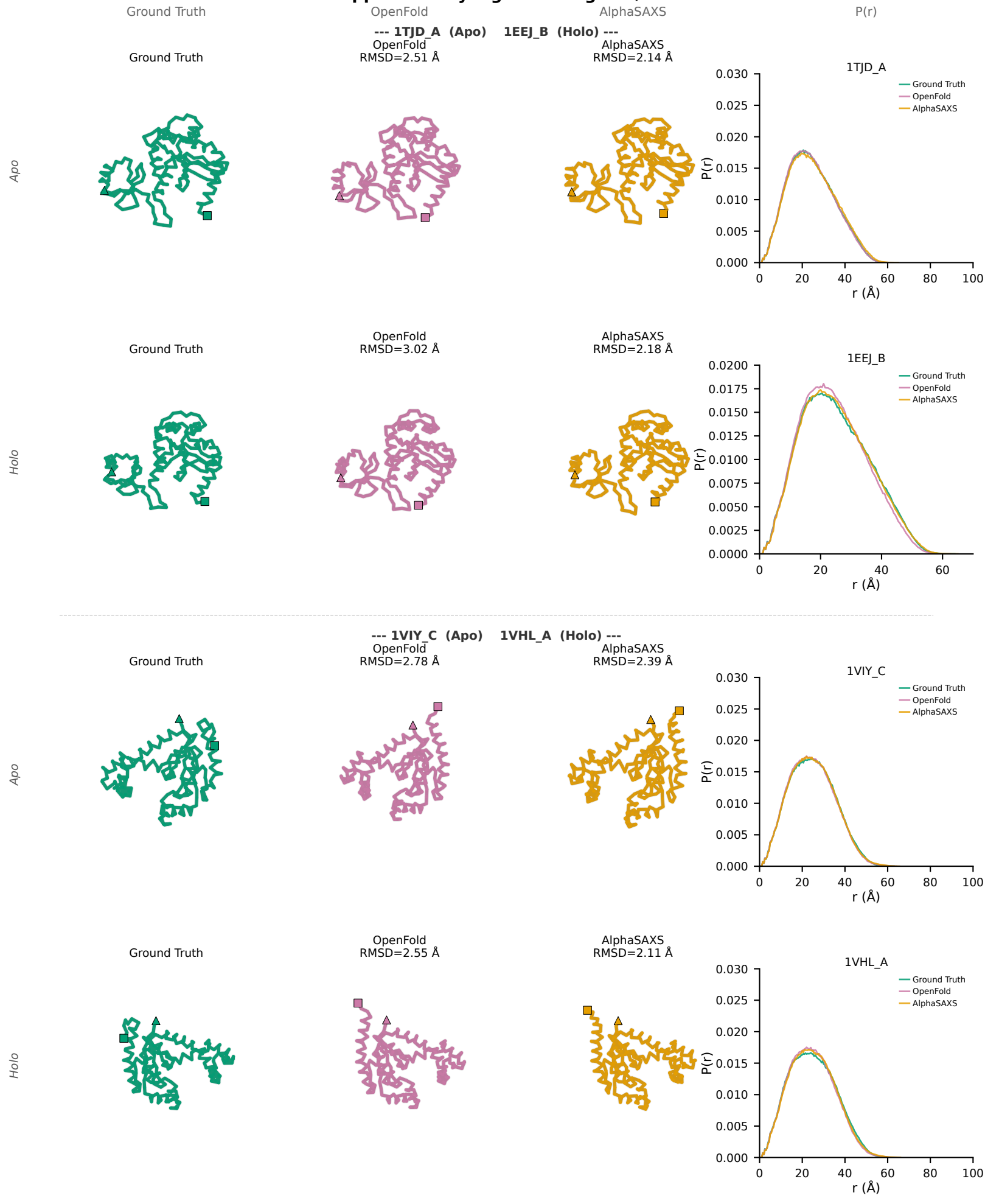
Ground Truth
OpenFold
AlphaSAXS
P(r)
--- 1TJD_A (Apo) 1EEJ_B (Holo) ---
Apo
Holo
Ground Truth
OpenFold
RMSD=2.51 Å
AlphaSAXS
RMSD=2.14 Å
1TJD_A
Ground Truth
OpenFold
AlphaSAXS
P(r)
r (Å)
Ground Truth
OpenFold
RMSD=3.02 Å
AlphaSAXS
RMSD=2.18 Å
1EEJ_B
--- 1VIY_C (Apo) 1VHL_A (Holo) ---
Ground Truth
OpenFold
RMSD=2.78 Å
AlphaSAXS
RMSD=2.39 Å
1VIY_C
Ground Truth
OpenFold
RMSD=2.55 Å
AlphaSAXS
RMSD=2.11 Å
1VHL_A

Ground Truth | OpenFold | AlphaSAXS | P(r)

--- 1W4U-9_A (Apo) 1UR6-4_A (Holo) ---

Apo

Ground Truth

OpenFold
RMSD=2.27 Å

AlphaSAXS
RMSD=2.31 Å

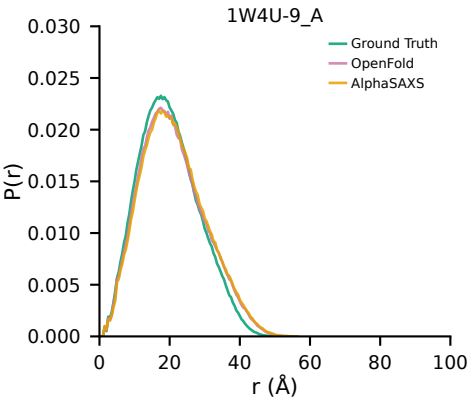

Holo

Ground Truth

OpenFold
RMSD=2.17 Å

AlphaSAXS
RMSD=2.32 Å

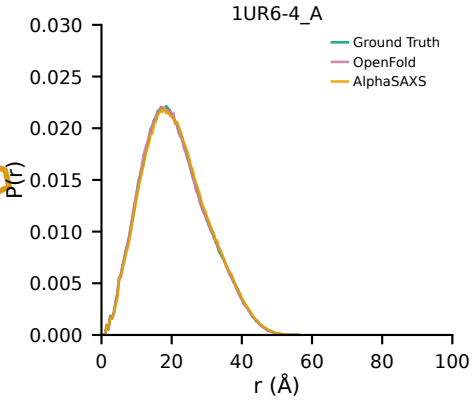

--- 1WD7_B (Apo) 1WCW_A (Holo) ---

Apo

Ground Truth

OpenFold
RMSD=5.10 Å

AlphaSAXS
RMSD=4.68 Å

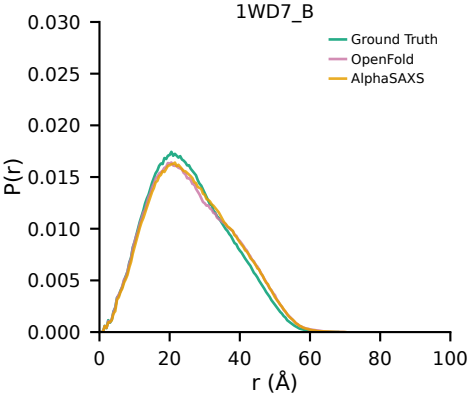

Holo

Ground Truth

OpenFold
RMSD=1.81 Å

AlphaSAXS
RMSD=1.54 Å

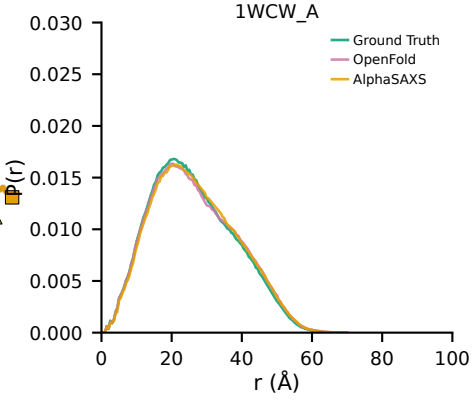

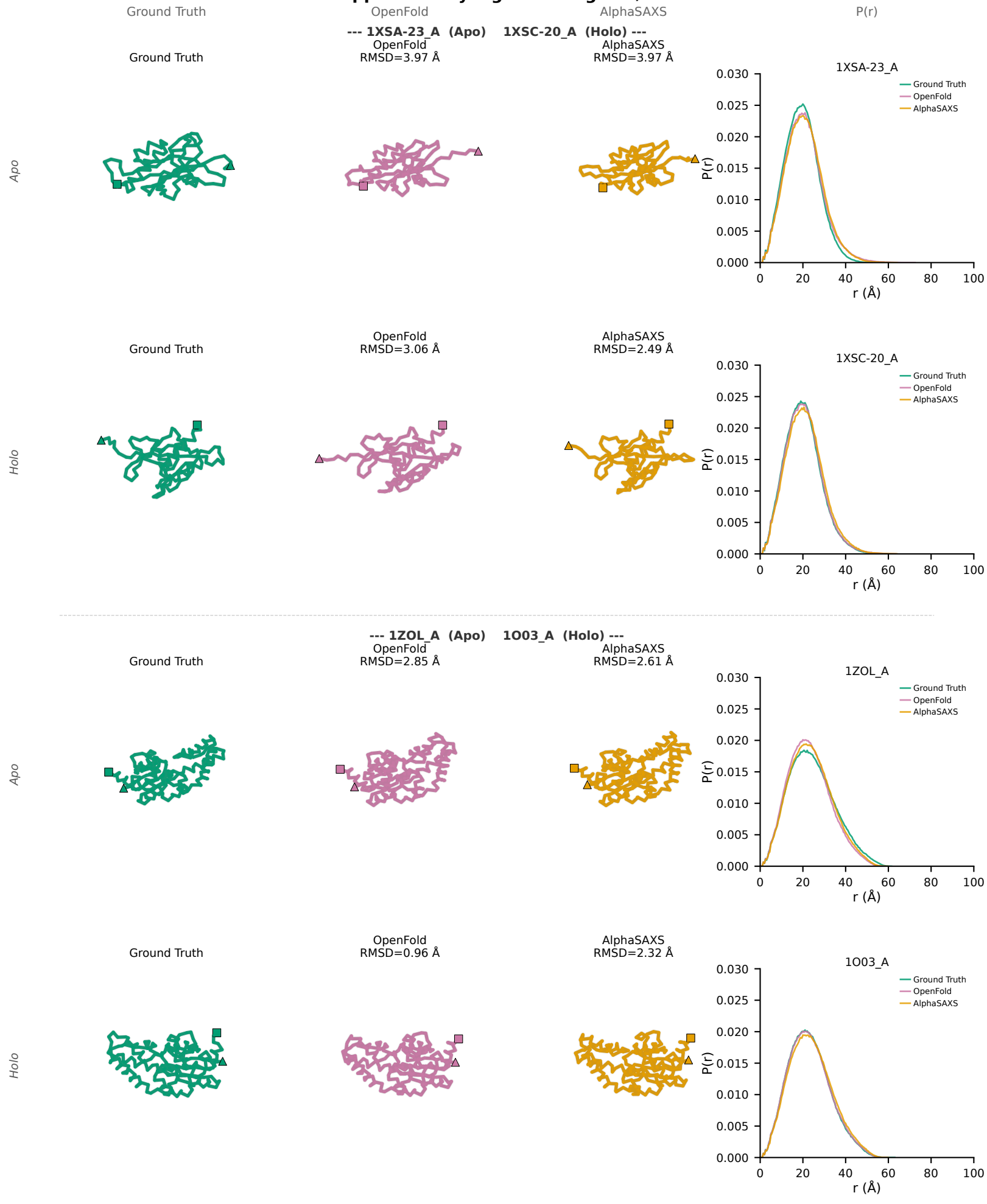
Ground Truth
OpenFold
AlphaSAXS
P(r)
--- 1XSA-23_A (Apo) 1XSC-20_A (Holo) ---
Apo
Ground Truth
OpenFold
RMSD=3.97 Å
AlphaSAXS
RMSD=3.97 Å
1XSA-23_A
Ground Truth
OpenFold
AlphaSAXS
P(r)
r (Å)
Holo
Ground Truth
OpenFold
RMSD=3.06 Å
AlphaSAXS
RMSD=2.49 Å
1XSC-20_A
--- 1ZOL_A (Apo) 1O03_A (Holo) ---
Apo
Ground Truth
OpenFold
RMSD=2.85 Å
AlphaSAXS
RMSD=2.61 Å
1ZOL_A
Holo
Ground Truth
OpenFold
RMSD=0.96 Å
AlphaSAXS
RMSD=2.32 Å
1O03_A

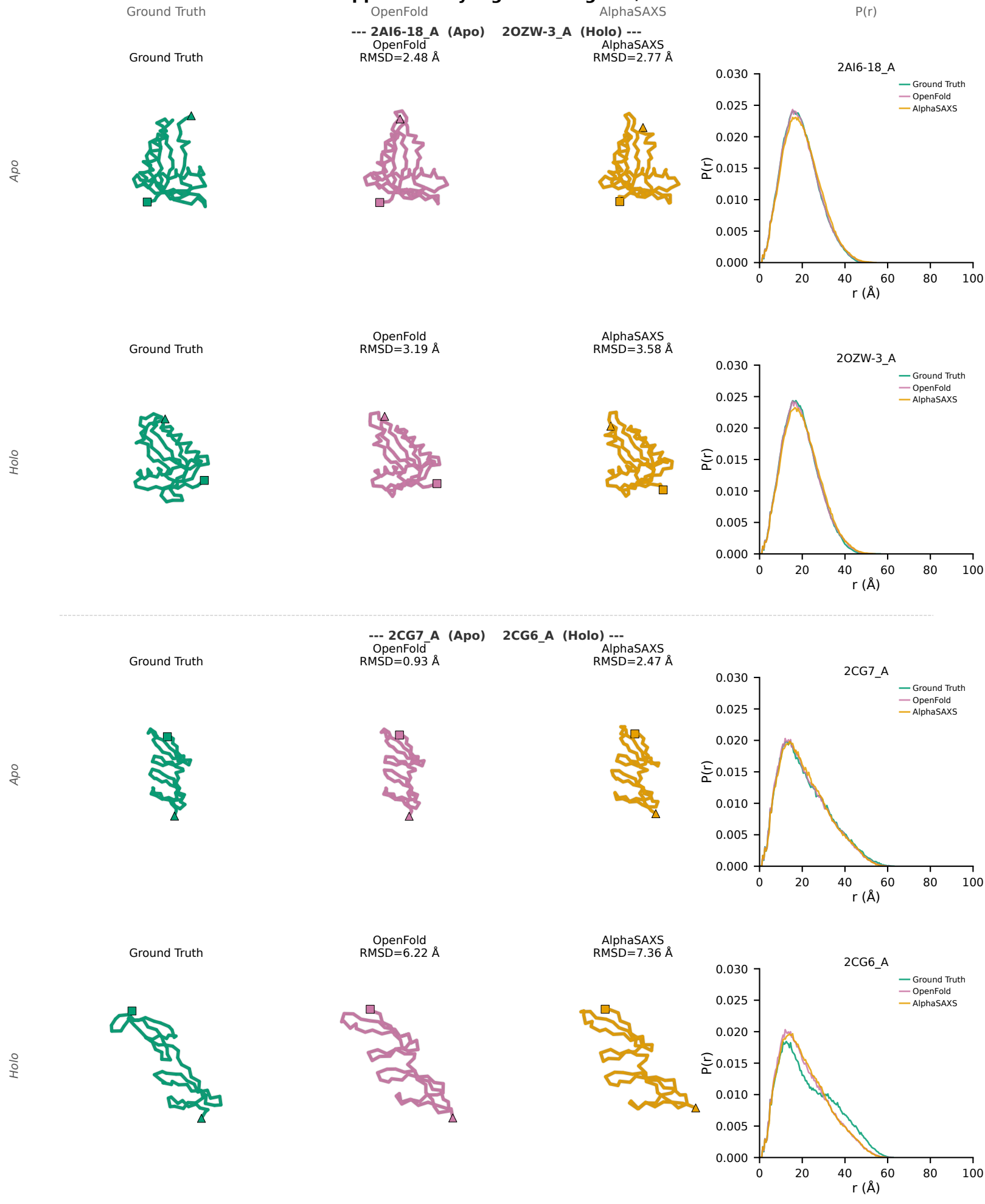
Ground Truth
OpenFold
AlphaSAXS
P(r)
--- 2AI6-18_A (Apo) 2OZW-3_A (Holo) ---
Apo
Holo
OpenFold
RMSD=2.48 Å
AlphaSAXS
RMSD=2.77 Å
2AI6-18_A
OpenFold
RMSD=3.19 Å
AlphaSAXS
RMSD=3.58 Å
2OZW-3_A
--- 2CG7_A (Apo) 2CG6_A (Holo) ---
OpenFold
RMSD=0.93 Å
AlphaSAXS
RMSD=2.47 Å
2CG7_A
OpenFold
RMSD=6.22 Å
AlphaSAXS
RMSD=7.36 Å
2CG6_A
Ground Truth
OpenFold
AlphaSAXS
P(r)
r (Å)

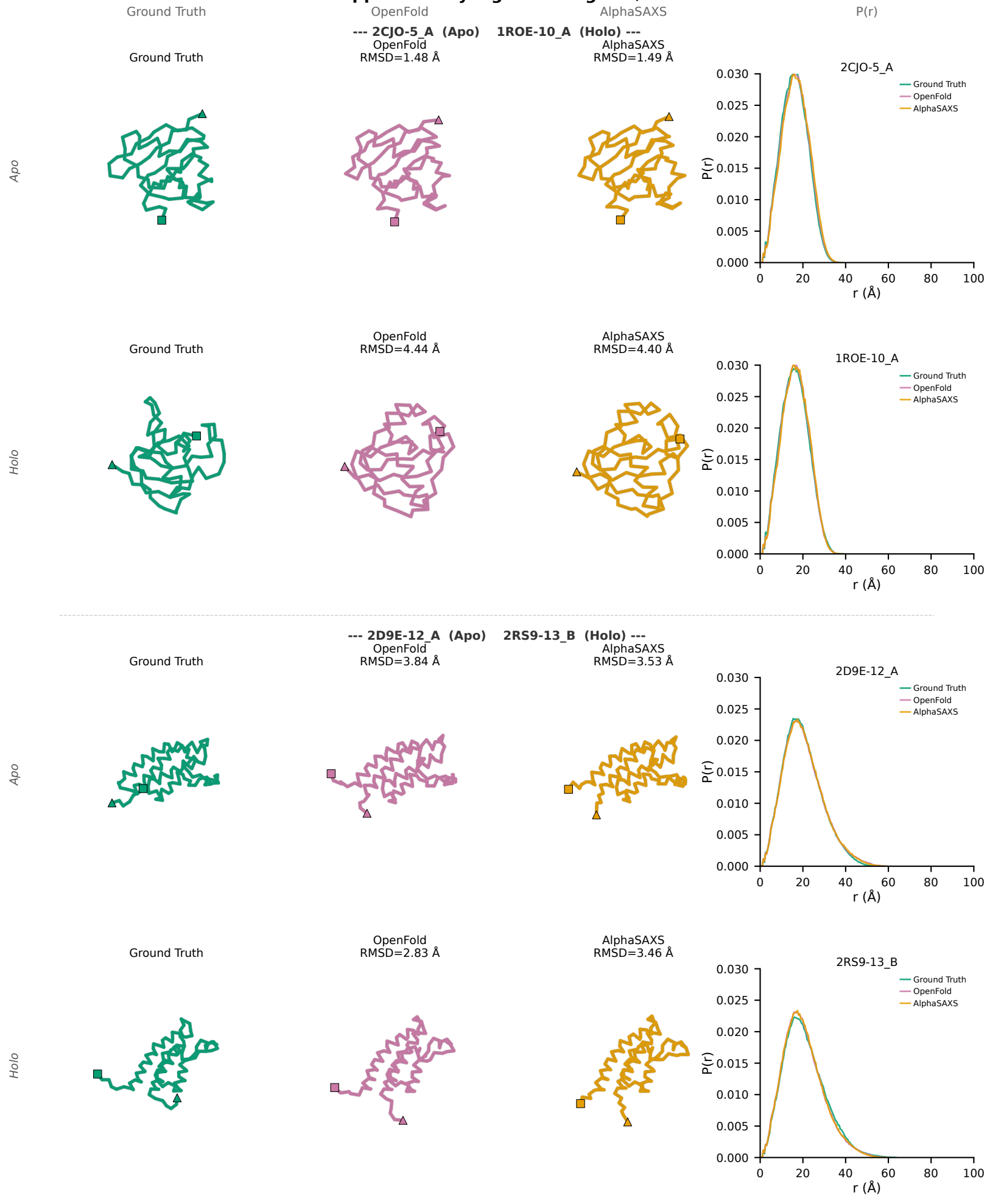
Ground Truth
OpenFold
AlphaSAXS
P(r)
--- 2CJO-5_A (Apo) 1ROE-10_A (Holo) ---
Apo
Holo
OpenFold
RMSD=1.48 Å
AlphaSAXS
RMSD=1.49 Å
2CJO-5_A
OpenFold
RMSD=4.44 Å
AlphaSAXS
RMSD=4.40 Å
1ROE-10_A
--- 2D9E-12_A (Apo) 2RS9-13_B (Holo) ---
OpenFold
RMSD=3.84 Å
AlphaSAXS
RMSD=3.53 Å
2D9E-12_A
OpenFold
RMSD=2.83 Å
AlphaSAXS
RMSD=3.46 Å
2RS9-13_B
Ground Truth
OpenFold
AlphaSAXS
P(r)
r (Å)

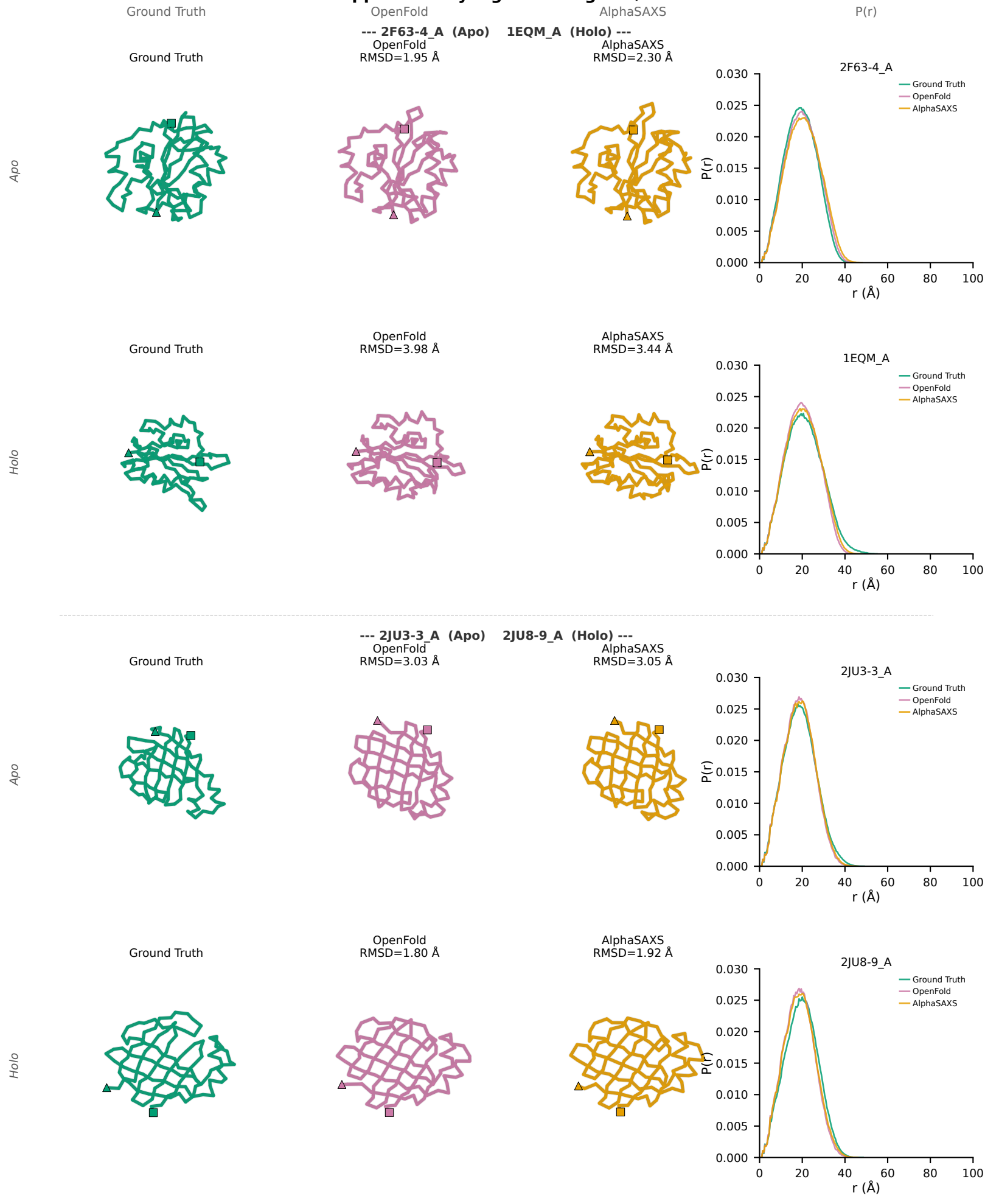
Ground Truth
OpenFold
AlphaSAXS
P(r)
--- 2F63-4_A (Apo) 1EQM_A (Holo) ---
Apo
Holo
OpenFold
RMSD=1.95 Å
AlphaSAXS
RMSD=2.30 Å
2F63-4_A
Ground Truth
OpenFold
AlphaSAXS
P(r)
r (Å)
OpenFold
RMSD=3.98 Å
AlphaSAXS
RMSD=3.44 Å
1EQM_A
--- 2JU3-3_A (Apo) 2JU8-9_A (Holo) ---
OpenFold
RMSD=3.03 Å
AlphaSAXS
RMSD=3.05 Å
2JU3-3_A
OpenFold
RMSD=1.80 Å
AlphaSAXS
RMSD=1.92 Å
2JU8-9_A

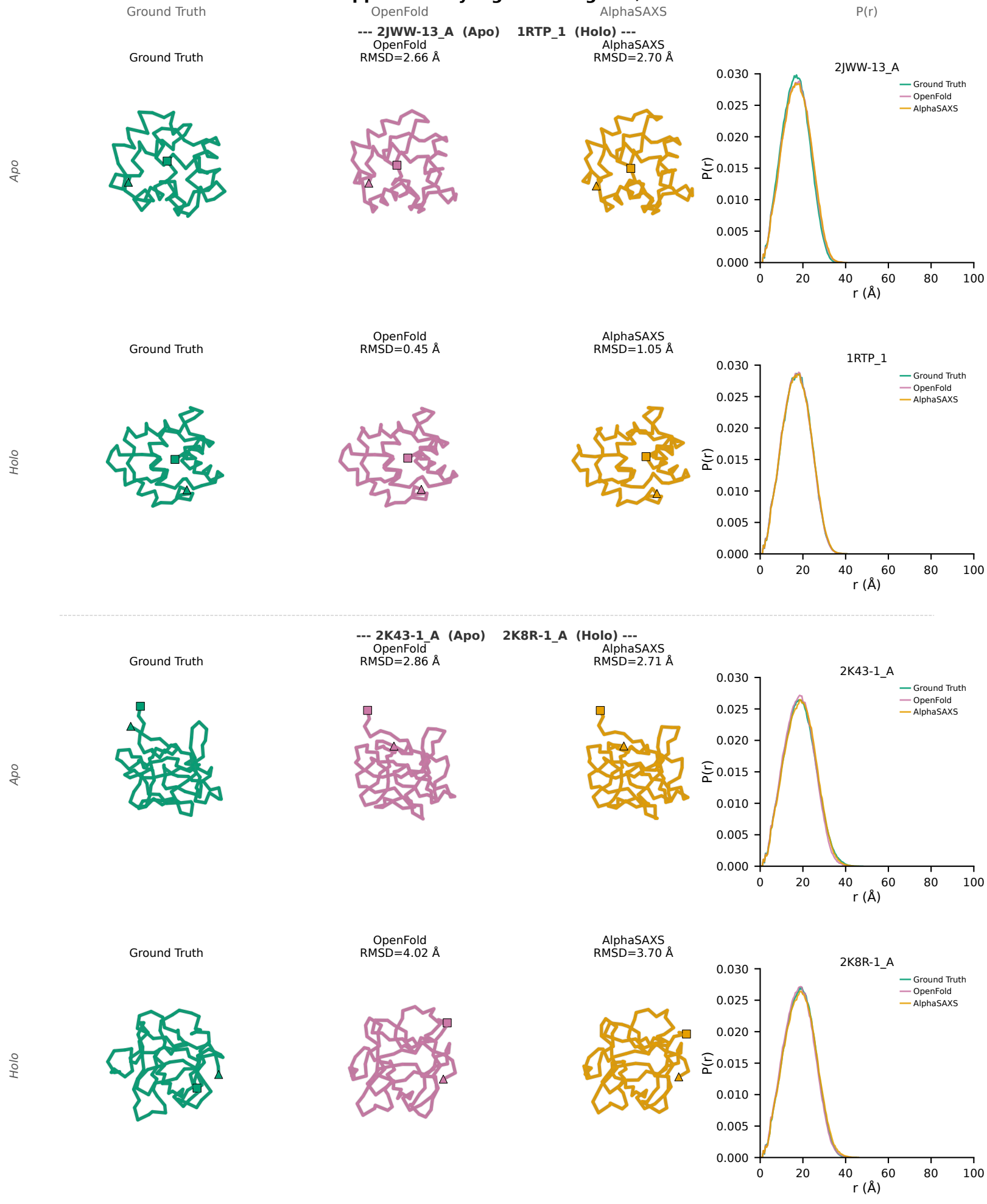
Ground Truth
OpenFold
AlphaSAXS
P(r)
--- 2JWW-13_A (Apo) 1RTP_1 (Holo) ---
Apo
Holo
Ground Truth
OpenFold
RMSD=2.66 Å
AlphaSAXS
RMSD=2.70 Å
2JWW-13_A
Ground Truth
OpenFold
AlphaSAXS
P(r)
r (Å)
0.030
0.025
0.020
0.015
0.010
0.005
0.000
0
20
40
60
80
100
Ground Truth
OpenFold
RMSD=0.45 Å
AlphaSAXS
RMSD=1.05 Å
1RTP_1
--- 2K43-1_A (Apo) 2K8R-1_A (Holo) ---
Ground Truth
OpenFold
RMSD=2.86 Å
AlphaSAXS
RMSD=2.71 Å
2K43-1_A
Ground Truth
OpenFold
RMSD=4.02 Å
AlphaSAXS
RMSD=3.70 Å
2K8R-1_A

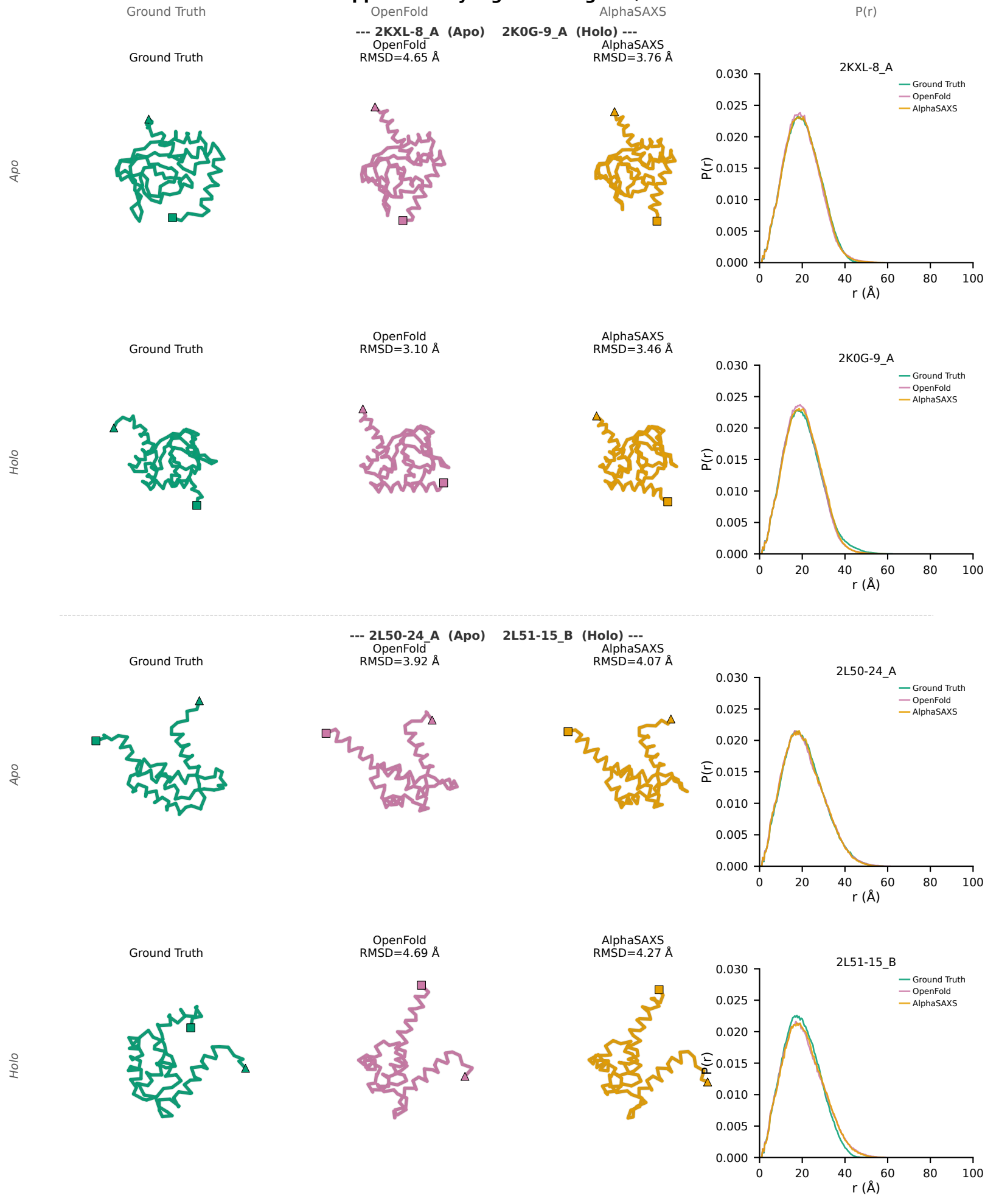
Ground Truth
OpenFold
AlphaSAXS
P(r)
--- 2KXL-8_A (Apo) 2K0G-9_A (Holo) ---
Apo
Holo
OpenFold
RMSD=4.65 Å
AlphaSAXS
RMSD=3.76 Å
2KXL-8_A
OpenFold
RMSD=3.10 Å
AlphaSAXS
RMSD=3.46 Å
2K0G-9_A
--- 2L50-24_A (Apo) 2L51-15_B (Holo) ---
OpenFold
RMSD=3.92 Å
AlphaSAXS
RMSD=4.07 Å
2L50-24_A
OpenFold
RMSD=4.69 Å
AlphaSAXS
RMSD=4.27 Å
2L51-15_B
Ground Truth
OpenFold
AlphaSAXS
P(r)
r (Å)

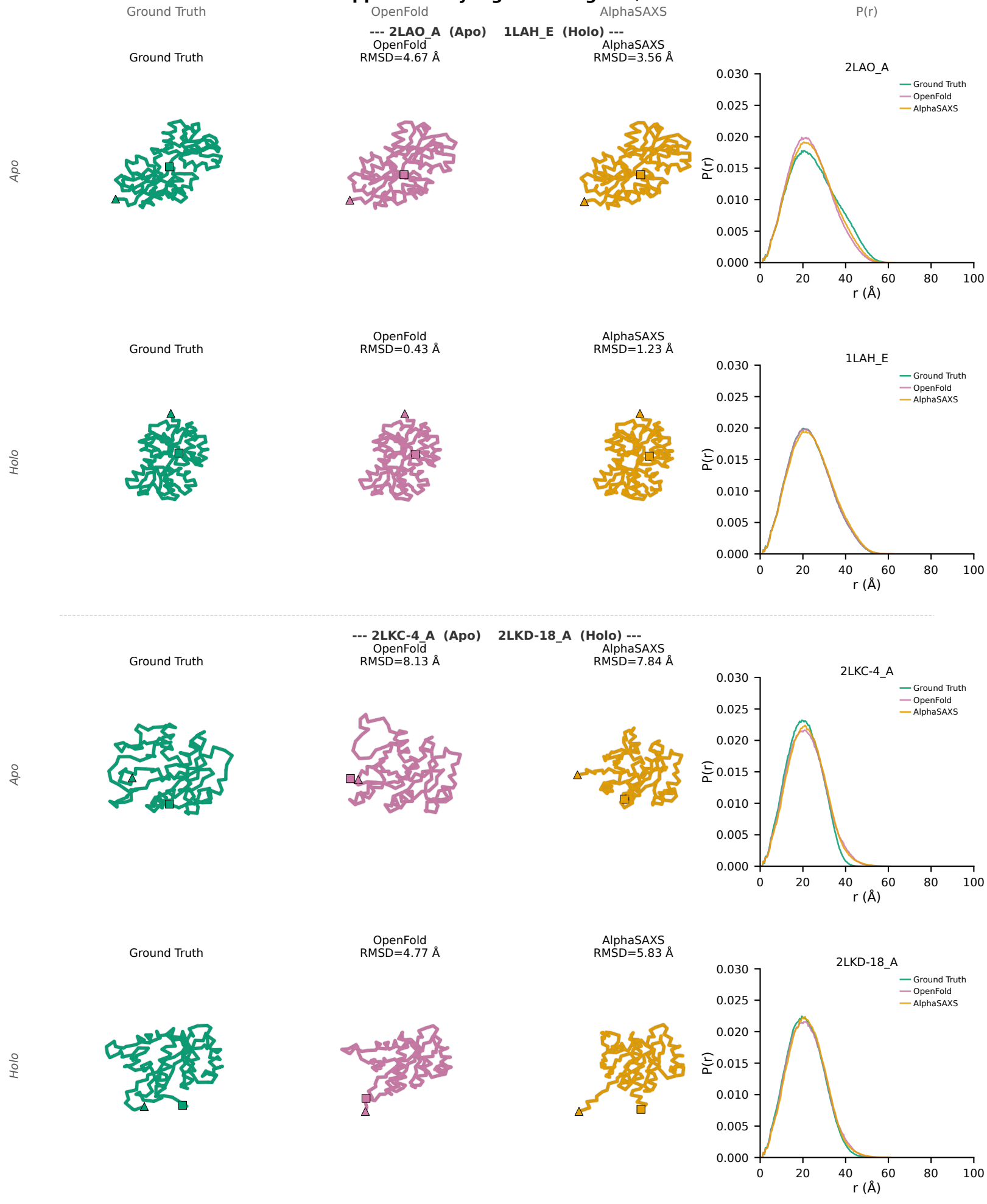
Ground Truth
OpenFold
AlphaSAXS
P(r)
--- 2LAO_A (Apo) 1LAH_E (Holo) ---
Apo
Holo
Ground Truth
OpenFold
RMSD=4.67 Å
AlphaSAXS
RMSD=3.56 Å
2LAO_A
Ground Truth
OpenFold
RMSD=0.43 Å
AlphaSAXS
RMSD=1.23 Å
1LAH_E
P(r)
r (Å)
--- 2LKC-4_A (Apo) 2LKD-18_A (Holo) ---
OpenFold
RMSD=8.13 Å
AlphaSAXS
RMSD=7.84 Å
2LKC-4_A
OpenFold
RMSD=4.77 Å
AlphaSAXS
RMSD=5.83 Å
2LKD-18_A

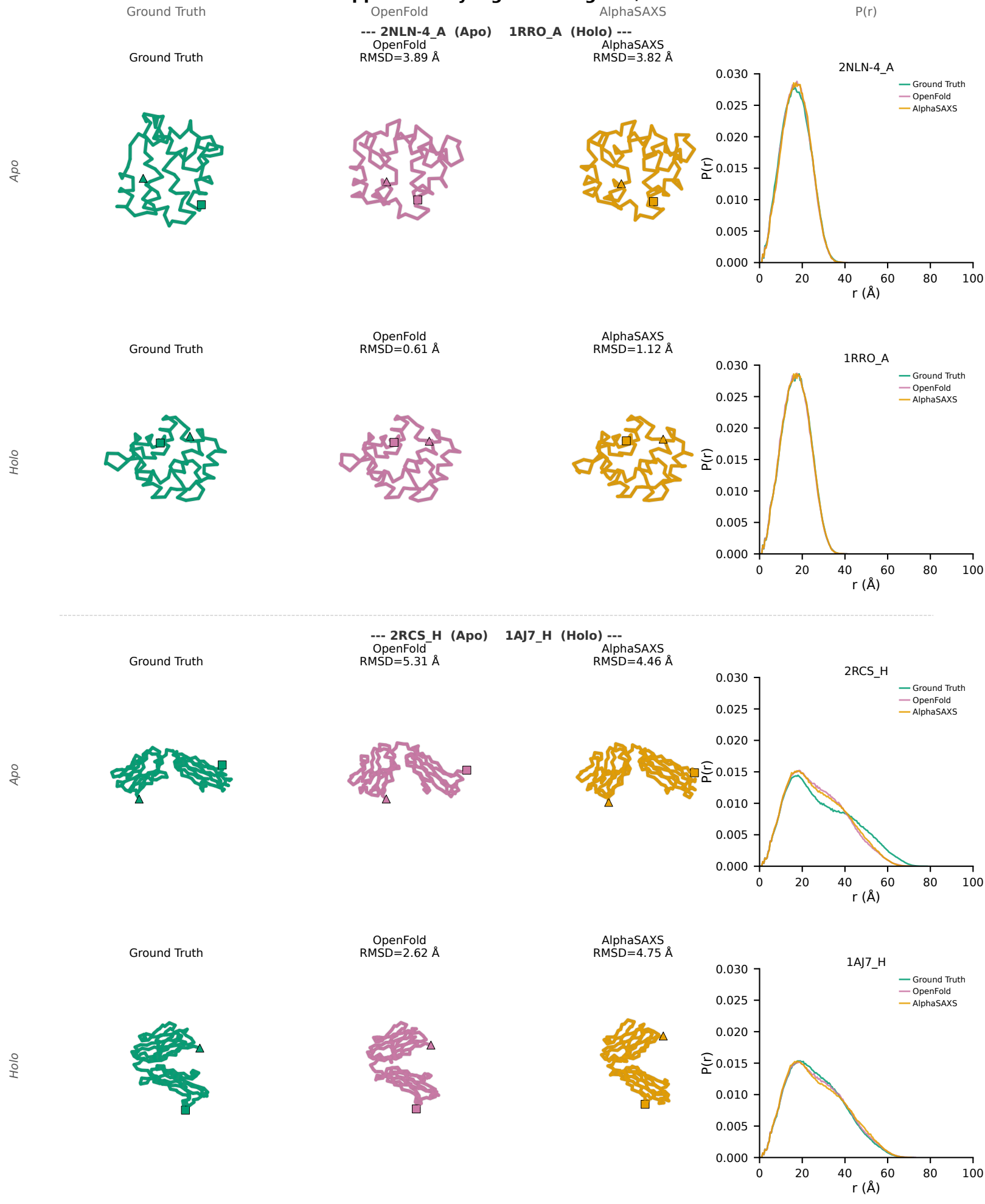
Ground Truth
OpenFold
AlphaSAXS
P(r)
--- 2NLN-4_A (Apo) 1RRO_A (Holo) ---
Apo
Holo
Ground Truth
OpenFold
RMSD=3.89 Å
AlphaSAXS
RMSD=3.82 Å
2NLN-4_A
Ground Truth
OpenFold
AlphaSAXS
P(r)
r (Å)
Ground Truth
OpenFold
RMSD=0.61 Å
AlphaSAXS
RMSD=1.12 Å
1RRO_A
--- 2RCS_H (Apo) 1AJ7_H (Holo) ---
Ground Truth
OpenFold
RMSD=5.31 Å
AlphaSAXS
RMSD=4.46 Å
2RCS_H
Ground Truth
OpenFold
RMSD=2.62 Å
AlphaSAXS
RMSD=4.75 Å
1AJ7_H

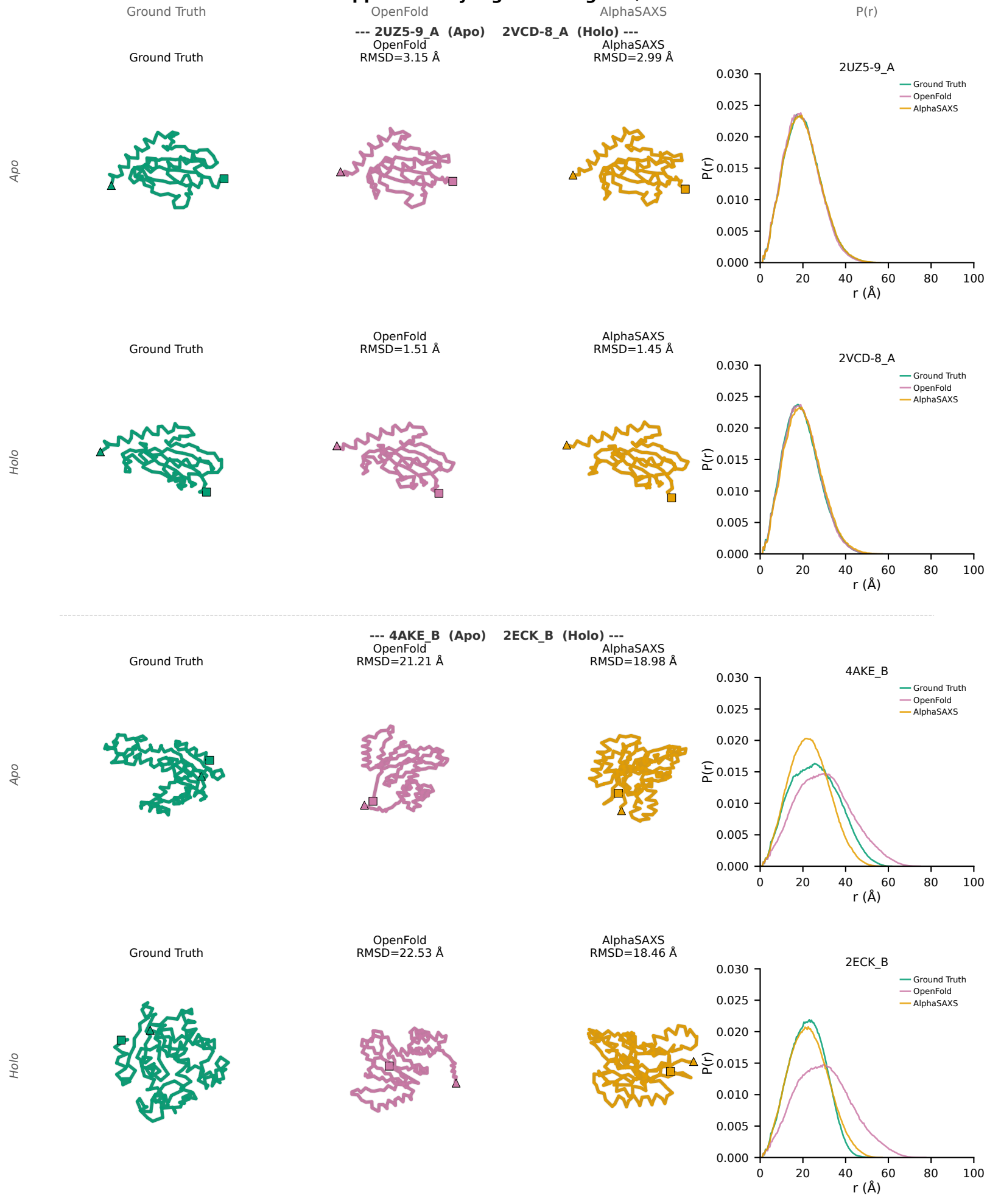
Ground Truth
OpenFold
AlphaSAXS
P(r)
--- 2UZ5-9_A (Apo) 2VCD-8_A (Holo) ---
Apo
Holo
Ground Truth
OpenFold
RMSD=3.15 Å
AlphaSAXS
RMSD=2.99 Å
2UZ5-9_A
Ground Truth
OpenFold
RMSD=1.51 Å
AlphaSAXS
RMSD=1.45 Å
2VCD-8_A
P(r)
r (Å)
--- 4AKE_B (Apo) 2ECK_B (Holo) ---
RMSD=21.21 Å
RMSD=18.98 Å
4AKE_B
RMSD=22.53 Å
RMSD=18.46 Å
2ECK_B
